# Changing Landscape of *Haemophilus influenzae* Meningitis and Implication on Public Health

**DOI:** 10.1155/2024/5571104

**Published:** 2024-04-24

**Authors:** John Allonce, Mohammed Ahsan, Angelina Browne, Rebecca Witherell, Mark Rasnake

**Affiliations:** NCH Healthcare System, Department of Internal Medicine, Naples, FL, USA

## Abstract

*Haemophilus influenzae* (*H. influenzae*) has evolved as a prominent pathogen, with nontypeable strains (NTHi) emerging as a leading cause of invasive disease, particularly among the elderly. Since the introduction of *Haemophilus influenzae* B (Hib) vaccine, invasive infection has shifted from children with Hib to the elderly with NTHi. NTHi affects those primarily with predisposing factors such as an immunocompromised state, CSF leakage, or ENT infections. We present two cases that emphasize the shift of invasive infection, risk factors, and elevated intracranial pressure (ICP) as a complication. *Case 1*. A 75-year-old female with a sudden onset of weakness and respiratory symptoms deteriorated rapidly. Imaging revealed mastoid effusion and ventriculitis, likely originating from otomastoiditis. Lumber puncture confirmed NTHi. ICU course complicated by elevated ICP prompted repeat lumbar puncture. The patient recovered after 8 days but not near baseline. *Case 2*. A 50-year-old female with altered mental status, headache, and ear pain exhibited signs of pansinusitis and pseudotumor cerebri. Elevated ICP was evident upon lumbar puncture, and NTHi was isolated in CSF and blood cultures. MRI of the brain showed prominent optic nerve sheaths and transverse sinus arachnoid granulations' concern for underlying pseudotumor cerebri. Repeat lumbar puncture or ventricular drainage was deferred after discussion with neurosurgery. Diabetes was identified as a comorbidity. The patient's condition improved after 14 days of antibiotics and dexamethasone. These cases emphasize the shifting landscape of *H. influenzae* meningitis, primarily driven by NTHi, especially among the elderly. Although NTHi infections were considered less invasive, recent epidemiology review indicated it as the leading cause of *H. influenzae* meningitis. With the increasing prevalence of NTHi and its increase in invasive patterns, it is crucial to implement vaccination strategies and develop new vaccines targeting NTHi.

## 1. Introduction

*Haemophilus influenzae* (*H. influenzae*) is a Gram-negative coccobacillus that is characterized by its encapsulated (typeable) and unencapsulated (nontypeable) forms. *H. influenzae* type B (Hib) was the leading cause of bacterial meningitis in infants prior to the introduction of the conjugate Hib vaccine [1]. Due to the widespread vaccine campaigns, the incidence of invasive *H. influenzae* disease has been found to be largely attributable to the nontypeable *H. influenzae* (NTHi) strains, particularly among the elderly (age > 65 years) [2–4].

The incidence of community-acquired meningitis secondary to *H. influenzae* in adults is 4% affecting those primarily with predisposing factors such as an immunocompromised state, cerebral spinal fluid (CSF) leakage, or ear, nose, and throat (ENT) infections [5]. The lack of an outer capsule makes NTHi strains inept in directly penetrating capillaries and thus the pathogenesis of meningitis secondary to NTHi is from direct entry into the central nervous system (CNS) via infection of the sinuses or the middle ear. We present a case series of *H. influenzae* meningitis presenting as sepsis complicated by increased intracranial pressure (ICP). These cases emphasize the evaluation of NTHi as the leading cause of severe invasive *H. influenzae* disease.

## 2. Case 1

A 75-year-old female presented with a chief complaint of generalized weakness with an onset of 2 hours. The patient describes having increased congestion and intermittent coughing for the past 4 days, along with episodes of emesis. She arrived at the emergency department with vitals notable for blood pressure of 188/88 mm·Hg, heart rate of 97 beats per minute, respiratory rate 23 breaths per minute, saturating 100% on room air, and temperature of 36.2°C. Initial labs were remarkable for lactate 3.2 mmol/L, procalcitonin 7.32 ng/ml, WBC 11.1/L, and potassium 3.2 mEq/L. A computed tomography (CT) head was done, which was negative for acute intracranial pathology, demonstrating chronic microvascular ischemia. Later that evening in the hospital wards, the patient became unresponsive, diaphoretic, with minimal movement of extremities. The patient had excessive drooling from the mouth and was promptly intubated and transferred to the ICU.

**Figure 1:**
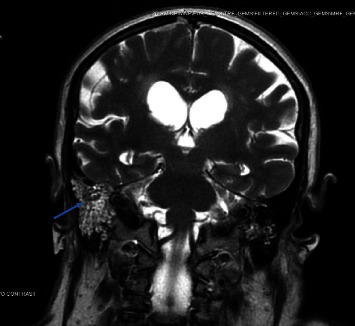
MRI of the brain (case 1) of a 75-year-old female right mastoid effusion with enhancement. Demonstrated by the blue arrow.

Due to change in the mental status, there was concern for meningitis complicated by sepsis; blood, sputum, and urine cultures were collected. The patient was started on empiric antibiotics including vancomycin, cefepime, ampicillin, and acyclovir. An MRI brain was ordered, which showed right mastoid effusion with enhancement, and developing ventriculitis likely secondary to the right otomastoiditis, as demonstrated in Figure 1. It was suspected that a sinus and airway infection progressed into a more systemic condition through hematogenous and direct inoculation. A lumbar puncture (LP) revealed opening pressure of 40 cm H20, PCR positive *H. influenzae*, glucose of 2 mg/dl, protein of 818 mg/dl, RBC 61,158 per mm^3^, and WBC 2418 per mm^3^ (with an 87% neutrophil predominance). Sputum and blood cultures were also positive for *H. influenzae*. Ear nose and throat (ENT) and infectious disease (ID) specialists were consulted; with ENT not recommending any surgical interventions. The patient was de-escalated off acyclovir, cefepime, vancomycin, and ampicillin and transitioned to ceftriaxone 2 g IV q12 h.

Due to the patient's elevated intracranial pressure, repeat LP was performed, with opening pressure improving to 27 cm of H_2_O. The patient's admission was complicated by episodes of A-fib RVR requiring rate control with metoprolol tartrate and diltiazem, and later being transitioned to amiodarone. The patient's neurological status gradually improved and was extubated 8 days after ICU admission. The patient was transitioned to the general floor with gradual improvement in encephalopathy. She was discharged to a skilled nursing facility with plans for 6 weeks of IV ceftriaxone. At 6 months, the patient continues with physical therapy but is managed by a non-network physician.

## 3. Case 2

A 50-year-old female presented for evaluation of altered mental status, left-sided headache, left ear pain, and eye discharge. On admission, temperature was of 37.9ºCelsius, heart rate 111 beats per minute, respiratory rate of 16 breaths per minute, blood pressure of 168/87 mm·Hg, and saturating 96% on room air. The patient's husband at bedside reported that the patient had upper respiratory symptoms for the past week with headaches and left ear pain. Laboratory findings were significant for WBC 22.2/L, potassium 3.1 mEq/L, and lactate 1.1 mmol/L. CT of the head showed chronic bilateral subdural hygromas, 2.6 left middle cranial fossa arachnoid cyst, and pansinusitis. An LP was performed with opening pressures of 55 cm of H_2_O and cloudy fluid. CSF studies revealed glucose 70 mg/dl, protein 269 mg/dl, WBC 2148 per mm^3^, and neutrophils 80% predominant. Blood and sputum cultures were obtained, and the patient was started on empiric therapy with cefepime, vancomycin, acyclovir, ampicillin, and dexamethasone. CSF PCR was positive for *H. influenzae* as well as human herpesvirus 6. Patient's blood cultures also returned positive for *H. influenzae.*

**Figure 2:**
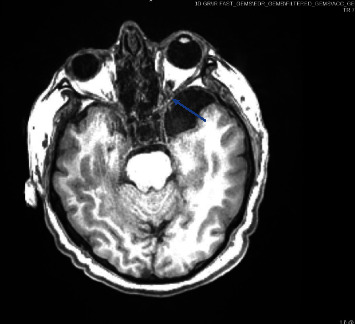
MRI of the brain of a 50-year old female with prominent optic nerve sheaths. Demonstrated by the blue arrow.

A brain MRI was ordered and did not demonstrate leptomeningeal enhancement but showed prominent optic nerve sheaths and transverse sinus arachnoid granulations, increasing concern for underlying pseudotumor cerebri, which is seen in Figure 2. Due to left eye conjunctivitis, an MRI orbit face neck was done, showing left middle cranial fossa arachnoid cyst, and subtotal opacification of the left ethmoid air cells, maxillary sinus, and sphenoid sinus. Patient's antibiotics transitioned to ganciclovir, ceftriaxone, and dexamethasone. Neurosurgery and ENT were consulted due to bilateral subdural hygromas and to evaluate the need for CSF diversion, but no intervention was recommended. The patient also started on erythromycin eye drops for her conjunctivitis. The patient was also found to have newly diagnosed type 2 diabetes mellitus, with an A1c of 13.2%. She was managed on insulin drip, and then transitioned to a basal-bolus insulin regimen. The patient continued to have improved mentation and was transferred to the medical floor. She continued to receive dexamethasone for a total of 5 days, and ceftriaxone 2 g IV twice a day for a total of 14 days. The patient's mentation improved to baseline, with no residual neurological, visual, or hearing deficits. The patient was discharged on an insulin regimen and metformin, with appropriate follow-up. At 6 month follow-up, the patient has intermittent olfactory hallucination (smells smoke) but is doing well. Diabetes is under control with an A1c of 6.6.

## 4. Discussion

*H. influenzae* can lead to infections such as pneumonia, otitis media, and even more invasive disease such as meningitis [6]. It significantly contributes to morbidity and mortality among both pediatric and geriatric populations, as well as individuals with specific underlying medical ailments [7]. *H. influenzae*-encapsulated strains tend to cause more invasive diseases, including bacteremia, meningitis, pneumonia, and epiglottitis. Hib is the most virulent strain, accounting for 95% of invasive diseases in children [8]. NTHi strains were known to be less invasive, causing common infections such as otitis media, sinusitis, and conjunctivitis [9]. With the introduction of Hib polysaccharide vaccine in 1985 and Hib conjugate vaccine, there was a significant decline in invasive Hib in children thereafter ([10, 11]). The decline of Hib has continued yearly to near disappearance with most cases occurring in unvaccinated or undervaccinated children. Since the introduction of Hib vaccine, we are now seeing increasing reports of *H. influenzae* disease caused by non-B serotypes and NTHi [12]. Although previous trends categorized NTHi as less invasive, recent epidemiology review indicated it as the leading cause of *H. influenzae* meningitis. A review of cases in 2009–2015, there was a mean annual incidence of invasive *H. influenzae* disease of 1.70 cases per 100,000 population. NTHi had the highest incidence (1.22) and case fatality (16%), as compared with Hib (0.03; 4%) and non-B-encapsulated serotypes (0.45; 11%) [13]. Risk factors of *H. influenzae* include those who are unvaccinated during childhood, particularly Hib, CSF leakage, ENT infections, advanced age, and immunocompromised state.

Specific predisposing factors for the patient in case 1 is her age exceeding 65 years. Studies detailing NTHi infections have indicated a notable prevalence within the age range of 65–74 years, with an escalated incidence of invasive infections observed as patients' age advanced [14]. In a review of 5991 cases of NTHi between 2008 and 2019, 93% of patients were admitted, and 37% required ICU level of care. 865 were over the age of 65 and diagnosed with bacteremia and meningitis [15]. This emphasizes the shift to a more invasive nature with patients over the age of 65 years being the most vulnerable. The patient in case 1 also had MRI findings of effusion in the right mastoid with complaints of decreased hearing during hospital examination. Otitis media frequently stems from *Streptococcus pneumoniae* succeeded by NTHi and *Moraxella catarrhalis*. The correlation between ENT infections and meningitis has been noted to reach 20%, establishing ENT as the most prominent risk factor for developing meningitis [16, 17]. This characteristic is further evident in both cases, with the second patient exhibiting MRI findings of opacification in the left ethmoid air cells, maxillary sinus, and sphenoid sinus. In case 2, risk factors also include immunocompromised state with undiagnosed/uncontrolled diabetes mellitus. Patients with diabetes are at higher risk of infection in general and this is evident as it is the fourth leading risk factor in patients who developed meningitis [17]. Due to the increase in NTHi and ongoing disease burden, it is essential we continue to monitor the shifts in invasive *H. influenzae* disease and begin to think and create public prevention strategies [7].

A common complication of bacterial meningitis is increased ICP. This complication is more often seen with other bacteria, in particular *Streptococcus pneumoniae* and *Neisseria meningitidis* rather than *H. influenzae* [18]. In selected patients with bacterial meningitis and clinical evidence of elevated ICP, monitoring and aggressive treatment to maintain cerebral blood flow could be crucial to survival [19]. In both cases, we see evidence of increased ICP resulting in further intervention, such as repeat imaging, repeat LP, additional specialist consults, or prolonged ICU care. In addition, in case 2, the presence of underlying pseudotumor cerebri exacerbated her elevated ICP. This potentially acted as a predisposing risk factor for meningitis. Case reports have noted a connection between idiopathic intracranial hypertension (IIH) and spontaneous CSF leakage, suggesting that her pseudotumor cerebri could predispose the patient to the development of meningitis [20]. Previous studies on the management of elevated ICP in bacterial meningitis suggest potential benefits in the utilization of continuous ICP monitoring. This advantage becomes more prominent in cases where clinical manifestations indicative of elevated ICP, such as pupillary dilation, are absent. Reports have shown decreased mortality and decreased sequela in those monitored for ICP and treated promptly, particularly in severe cases. Further exploration into diverse modalities for monitoring ICP in patients with bacterial meningitis and elevated ICP is needed [21, 22].

The incidence of invasive NTHi primarily in patients over 65 years in the United States is on the rise. This could be due to waning immunity due to the absence of booster vaccines, decreased childhood vaccination, or the presence of other comorbidities [23]. Current guidelines regarding Hib vaccine per CDC recommend routine childhood immunization schedules. There is no current recommendation for routine Hib booster or revaccination in adults as the rate of invasive Hib significantly decreases with age. CDC only recommends vaccination in adults with no prior history of Hib vaccine if there is a history of elective splenectomy, functional or anatomic asplenia, or recipients of hematopoietic stem cell transplant. After the Hib vaccine was introduced, it has been observed that it does not have any impact on infections caused by NTHi [24]. As a result, the prospect of administering additional doses or boosters of the Hib vaccine to individuals over the age of 65 years, in reducing the severity of invasive NTHi infections, appears less necessary. The introduction of the vaccine does create a selective survival of NTHi which would explain such an increase in diagnosis. The elimination of Hib in the nasal pharynx and upper airway has led to a predominant colonization of NTHi. Presently, there is an ongoing inquiry into the potential development of a vaccine targeting NTHi. Such a vaccine would hold a significant value, particularly considering the increasing incidence of invasive NTHi cases in comparison to other variants of invasive *H. influenzae*. However, the advancement of an NTHi vaccine remains complex due to the inherent heterogeneity of NTHi, which poses challenges in identifying immunogenic antigens suitable for generating a broad-spectrum antibody response [25]. Currently, there is investigation into a vaccine potential against HMW1 and HMW2. These are adhesion proteins found with NTHi. If damaged, it may decrease NTHi adhesion to the nasal pharynx thereby decreasing colonization. The investigation is early but appears promising [26].

Furthermore, given how NTHi tends to integrate within biofilms with other pathogens, another intervention being investigated is the use of monoclonal antibodies to interrupt this process. Studies have shown that two monoclonal antibodies targeting a subunit specific to NTHI (PilA) and to DNABII (common structural protein in bacteria) led to a breakdown of the biofilm, allowing for more effectiveness of antibiotics. Further research should also investigate the role of using monoclonal antibodies, as it may allow for early eradication and prevention of serious infections such as meningitis seen in our case [27].

In conclusion, we highlight the changing nature of *H. influenzae* meningitis, with a focus on the NTHi and their clinical complexities. The cases we presented emphasize the importance of NTHi as a major cause of invasive *H. influenzae* disease, particularly among the elderly population. These cases also demonstrate the potential severity of NTHi meningitis, which can lead to complications such as elevated intracranial pressure (ICP), invasive management, and extended hospital stays. It is crucial to continue monitoring the changing pattern of *H*. *influenzae* and develop new vaccines targeting NTHi to address the challenges presented by this constantly evolving pathogen.

## Data Availability

The data used to support the findings of this study are included within the article.
